# Small Thaw Ponds: An Unaccounted Source of Methane in the Canadian High Arctic

**DOI:** 10.1371/journal.pone.0078204

**Published:** 2013-11-13

**Authors:** Karita Negandhi, Isabelle Laurion, Michael J. Whiticar, Pierre E. Galand, Xiaomei Xu, Connie Lovejoy

**Affiliations:** 1 Centre for Northern Studies (CEN) and Institut national de la recherche scientifique, Centre Eau Terre Environnement, Quebec, Canada; 2 School of Earth and Ocean Sciences, University of Victoria, Victoria, British Columbia, Canada; 3 UPMC Université Paris 06, (UMR 8222, LECOB), Observatoire Océanologique, Banyuls-sur-mer, France; 4 CNRS, UMR 8222, LECOB, Observatoire Océanologique, Banyuls-sur-mer, France; 5 Department of Earth System Science, University of California Irvine, Irvine, California, United States of America; 6 Département de biologie, Institut de Biologie Intégrative et des Systèmes, Université Laval, and Takuvik (CNRS, UMI 3376), Quebec, Canada; Dowling College, United States of America

## Abstract

Thawing permafrost in the Canadian Arctic tundra leads to peat erosion and slumping in narrow and shallow runnel ponds that surround more commonly studied polygonal ponds. Here we compared the methane production between runnel and polygonal ponds using stable isotope ratios, ^14^C signatures, and investigated potential methanogenic communities through high-throughput sequencing archaeal 16S rRNA genes. We found that runnel ponds had significantly higher methane and carbon dioxide emissions, produced from a slightly larger fraction of old carbon, compared to polygonal ponds. The methane stable isotopic signature indicated production through acetoclastic methanogenesis, but gene signatures from acetoclastic and hydrogenotrophic methanogenic Archaea were detected in both polygonal and runnel ponds. We conclude that runnel ponds represent a source of methane from potentially older C, and that they contain methanogenic communities able to use diverse sources of carbon, increasing the risk of augmented methane release under a warmer climate.

## Introduction

**Figure 1:**
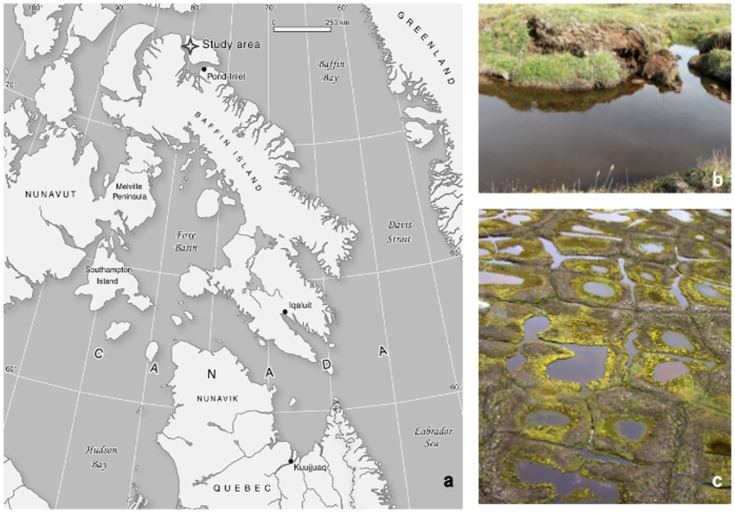
Study site description. (**a**) Map indicating the location of the study site on Bylot Island, Sirmilik National Park, Nunavut, Canada, (**b**) collapsed peat polygon ridges forming runnel ponds, and (**c**) landscape combining runnel and polygonal ponds.

In arctic regions, the acceleration of permafrost thaw and deepening of the seasonal active layer leads to thaw pond formations due to the organic and ice-rich ground subsiding [1]–[2]. These thaw ponds are also sometimes referred to as thermokarst lakes, since they superficially resemble ponds formed by the dissolution of limestone (karst). Two main geomorphological forms are commonly found in continuous permafrost regions of Eastern Canada: (i) small, shallow, narrow runnel ponds formed over melting ice wedges where peat slumping occurs, and (ii) more stable, slightly larger and deeper, polygonal ponds, which are naturally linked to the active layer freeze-thaw cycles, and can be colonized by aquatic plants and microbial mats (Fig. 1). Greenhouse gas (GHG) emissions from thermokarst ecosystems are highly variable [3]–[5] and often not considered in large-scale GHG studies and global carbon cycling models since small ponds cannot be seen with remote sensing tools [6]–[7]. These ponds have the potential to be significant GHG emitters contributing to a positive carbon-climate feedback [8]–[10], attributed to the mobilization of old stored carbon (C) stocks released back into the atmosphere [11]–[13]. In these ecosystems, microbial decomposers and methanogens have access to large quantities of allochthonous organic matter [4], [14]. The CH_4_ released from Siberian thaw lakes is significant and originates from microbial utilization of C stocks deposited thousands of years ago [8], [15]. In the eastern Canadian Arctic, C deposition dates from the Holocene [1], but microbial utilization is unknown.

The conversion of organic C previously locked in permafrost to GHG is highly dependent on its lability to microbial degradation [12], [16], [17]. For instance, fresh labile organic matter favors acetoclastic methanogenesis (AM) [18], where the organic substrate (i.e. acetate, methanol, methylated substrates, etc.) is cleaved and the methyl group is reduced to CH_4_. Comparatively, more recalcitrant compounds leached from peat favors the hydrogenotrophic pathway (HM) [19], which utilizes H_2_ to reduce CO_2_. Therefore, the available substrate selectively determines the methanogenic community and CH_4_ production rate.

Once CH_4_ is produced through AM or HM pathways, at the bottom of lakes and ponds, it is transported through the water column to the atmosphere by diffusion and ebullition. Ebullition transport can be classified as background, point sources or hotspots. In Siberian thermokarst lakes these three sources accounted for 25, 58, and 12% respectively, with the remaining 5% of total emissions attributed to diffusion [20]. Diffusion is generally considered less important than ebullition [21]–[22]. However, diffusion and ebullition rates are variable in aquatic systems and relative contribution of these sources has not been investigated in other Arctic thermokarst systems where geomorphology varies considerably. There are no previous reports from runnel type ponds and their potential contribution to atmospheric GHG is not known.

The objective of our study was to evaluate the release and potential for GHG emissions in the poorly studied runnel ponds compared with polygonal ponds of northeastern Canada. These ponds have the potential to form in ice and organic rich soils of permafrost and glaciated-influenced landscapes, covering ca. 9.6 million km^2^ of the global northern landscape [23]. The methanogenic pathways and C age were investigated through stable isotopic signatures and radiocarbon dating of dissolved and bubbling GHG. Archaeal community composition in the sediments was analyzed with high-throughput 16S rRNA gene pyrosequencing. We found that runnel ponds were supersaturated in CO_2_ and had more than 3 folds greater CH_4_ emissions than polygonal ponds, which were a CO_2_ sink. Higher CH_4_ emission is likely explained by a higher supply of organic matter under more hypoxic conditions, where CH_4_ oxidation is less likely to occur. The methanogenic community included genera capable of both AM and HM, indicating that methanogens could potentially use different carbon substrates and thus acclimate to changing conditions, for example vegetation cover or hydrology, under a warmer climate.

## Results

### Pond limnological properties

Within the four ponds targeted for the archaeal diversity study, runnel ponds, which are subjected to more peat leaching and erosion, had higher concentrations of DOC, nutrients (TN, SRP and TP) and iron (Table 1). Polygonal ponds showed no sign of recent erosion, with thick cyanobacteria-dominated microbial mats on the bottom, and lower concentrations of DOC, nutrients and ions. The organic carbon (OC) content of surface sediment was highly variable, ranging between 1.0 and 25.1% among the series of sampled ponds (n = 26, 2011), and with no significant difference (paired t-test) between polygonal ponds (8.4±6.6%) and runnel ponds (6.4±3.9%). Over the year, pond ice and water temperatures ranged between −26.7 and +21.4°C (averaging −7.6°C; Figure S1 in File S1). The temperature records showed that ponds remained frozen from ∼25 September to 4 June.

**Table 1:** Surface water physicochemical properties of the four ponds sampled for archaeal communities between 19 and 26 July 2009, including dissolved organic carbon (DOC, mg L^−1^), soluble reactive phosphorus (SRP, µg L^−1^), total phosphorus (TP, µg L^−1^), total nitrogen (TN), nitrate (NO_3_), sulfate (SO_4_), iron (Fe) all in mg L^−1^, pH, and dissolved CO_2_ and CH_4_ concentrations, both in µM.

POND	DOC	SRP	TP	TN	NO_3_	SO_4_	Fe	pH	CO_2_	CH_4_	OC
**Polygonal ponds**											
**BYL1**	8.4	<0.2	15.6	363	0.05	1.47	0.299	8.7	6.3	1.0	4.6
**BYL22**	8.1	<0.2	25.5	371	0.04	0.85	0.557	7.2	25.0	1.9	5.3
**Runnel ponds**											
**BYL24**	11.5	1.0	25.5	398	0.04	0.67	1.012	7.1	33.0	3.4	6.5
**BYL27**	11.8	0.5	26.3	822	0.06	1.56	0.905	6.6	78.8	2.6	17.8

Surface sediment organic carbon content (OC as percent) samples were collected between 12 June and 15 July 2011.

### GHG concentrations, fluxes and isotopic signatures

Surface water GHG concentrations collected in the compiled series of thaw ponds (from 2009 to 2011, n = 91) showed a significantly higher concentration of CH_4_ in runnel compared to polygonal ponds (t-test, df = 90, p = 0.003). Only runnel ponds were supersaturated in CO_2_ (averaging 119±124 µM, compared to polygonal ponds 9.6±8.9 µM) but all ponds were supersaturated in CH_4_ (4.1±4.7 and 1.3±1.7 µM, in runnel and polygonal ponds respectively). Runnel ponds also had significantly higher CO_2_ and CH_4_ fluxes compared to polygonal ponds (p≤0.0001; Figure S2 in File S1) but the diffusive flux of CO_2_ (−8.1 to 76.9 mmol m^−2^ d^−1^) and CH_4_ (0.02 to 6.3 mmol m^−2^ d^−1^) varied greatly over the 3 sampled summers. In the two ponds (BYL80 and BYL1) that were tested over 26 hours, diurnal dissolved GHGs varied by 18 and 25% for CO_2_, and 17 and 21% for CH_4_. The corresponding fluxes likely varied by no more than 25% throughout a day as estimated using a wind-based model incorporating the wind speed over the preceding 2 h,where the coefficient of variation was 45% for the wind speed. In the same two polygonal ponds (BYL1 and BYL80), eight separate measurements of CH_4_ ebullition fluxes showed that despite the variability within ponds, fluxes were always greater in BYL80 than in BYL1 (t-test, df = 7, p = 0.02, Table S1in File S1). Ebullition flux was lower than diffusive flux in BYL1 (representing on average 27% of total CH_4_ emission) and higher than diffusive flux in BYL80 (82% of total emission). The diffusive flux values used in this comparison were from approximately the same period in 2011, but ebullition was calculated over up to 32 h of bubble collection, while diffusive flux was always estimated from one discrete gas sample. The CH_4_ concentration in bubbles was also variable (1.5–32% by volume).

**Figure 2:**
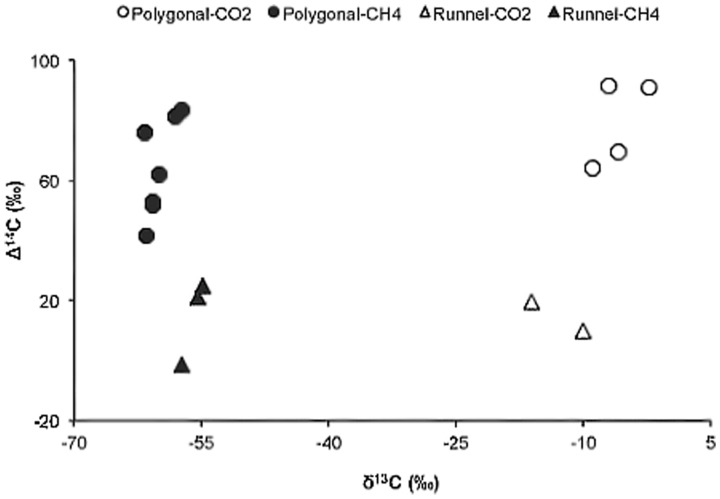
CH_4_ and CO_2_ carbon source and age. Radiocarbon signature (Δ^14^C) plotted against δ^13^CH_4_ and δ^13^CO_2_ showing: 1) that as the fraction of young carbon becomes higher for both CH_4_ and CO_2_, the δ^13^C signatures become more divergent indicating a decoupling in carbon source; 2) the runnel ponds CH_4_ contains a higher fraction of old carbon.

**Figure 3:**
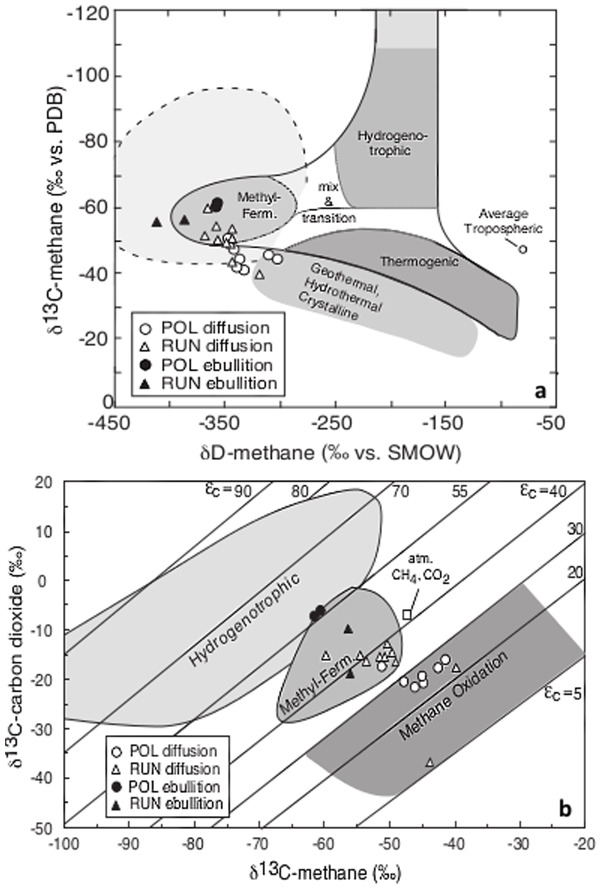
Methane production pathway through stable isotopes. (**a**) δ^13^CH_4_ against δD_CH4_ signatures of diffusive (2009) and ebullition (2011) CH_4_, indicating that acetoclastic methanogenesis (AM) is the dominant pathway in polygonal and runnel thaw ponds for samples collected in June/July. (**b**) δ^13^CO_2_ against δ^13^CH_4_ in thaw ponds showing the predominance of acetoclastic methanogenesis (AM) and the methanotrophic oxidation level for dissolved and ebullition CH_4_.

Overall the Δ^14^C signatures of the GHG released from both polygonal and runnel ponds through ebullition (−1.1 to 114.9‰) were categorized as modern (within the last ∼60 years). However, ebullition CH_4_ from two runnel ponds (n = 3) contained a higher fraction of old C compared to the two polygonal ponds (n = 7; p = 0.002; Fig. 2). Both C and H stable isotopic signatures indicate that all CH_4_ emitted by diffusion and ebullition during summer was produced from AM (Fig. 3a). The possibility of HM, as seen in Fig. 3b, was ruled out with the inclusion of δD-CH_4_ signature. There was no significant difference in the δ^13^C-CO_2_ or δ^13^C-CH_4_ values between the polygonal and runnel ponds (Table S2 in File S1), supporting the idea of similar methanogenesis production pathways (AM). However, there were indications that the CH_4_ emitted by diffusion was more susceptible to oxidation in the polygonal ponds (Fig. 3b). In fact, there was a significant relationship between the oxygen concentration at the surface of ponds and δ^13^C-CH_4_ (r^2^ = 0.337; p = 0.009). Comparatively, CH_4_ emitted through ebullition showed no signs of oxidation.

### Archaeal assemblages

**Figure 4:**
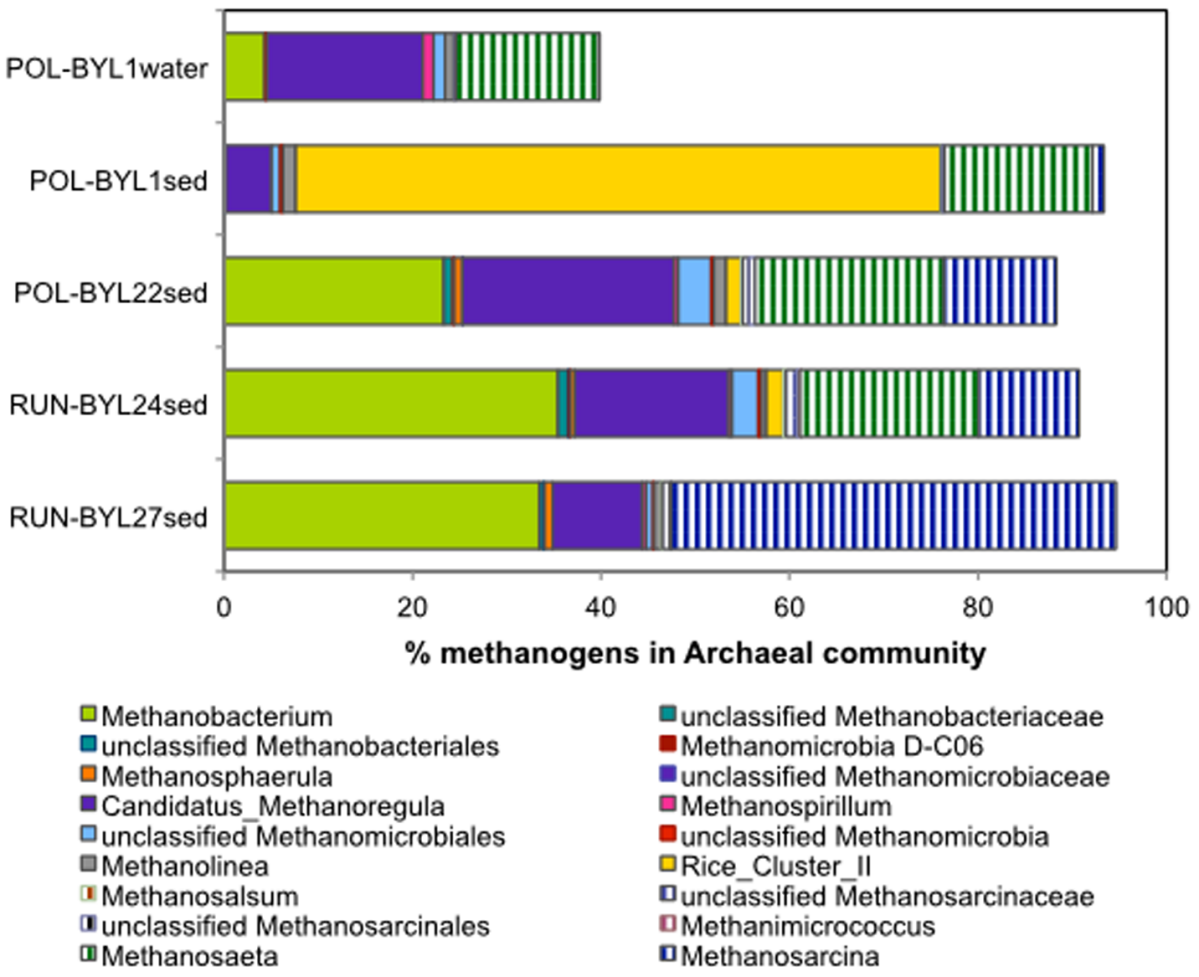
Archaeal methanogenic community of thaw ponds. Methanogen taxa retrieved from the sediment of four Arctic thaw ponds and from one water sample. Checkered symbols represent AM and solid are HM.

There was a predominance of methanogen 16S rRNA sequences in surface sediment archaeal communities in the 4 ponds (88–95% of the sequences). In contrast, the methanogens represented only 40% of the sequences in the water community of one polygonal pond where we were able to amplify the 16S rRNA gene. We also failed to amplify sediment DNA from one runnel pond (BYL38). The poor PCR success may have been due to a lack of Archaeal template present in the water samples, but is unexplained for BYL38 sediment sample. The water sample archaeal communities were dominated by sequences belonging to the uncultured clusters LDS and RCV (Fig. 4), and no anaerobic methanotrophic archaea were detected. The sediment non-methanogenic OTUs (5–12% of the sequences) belonged to the phylum Euryarchaeota, mainly of a terrestrial miscellaneous euryarchaeotal group (TMEG), and from the miscellaneous crenarchaeotic group (MCG). As there are no cultivated representatives of these groups, the metabolism of these environmental clusters is not known.

Altogether the majority of Archaea operational taxonomic units (OTUs) from surface sediments were classified into the four known methanogenic genera (*Methanobacterium*, Candidatus *Methanoregula*, *Methanosarcina* and *Methanosaeta*) and one uncultured group (Rice cluster II, RC II) within the deeply branching *Methanomicrobiales* (Fig. 4). Overall, the polygonal pond sediments were dominated by archaeal sequences belonging to HM (*Methanobacterium*, *Methanoregula*) representing from 63 to 82% of the putative methanogen sequences, while runnel ponds were either dominated by HM (65%, BYL24) or by AM (*Methanosarcina, Methanosaeta*) (51%, BYL27). The most abundant *Methanobacterium* that represented one third of all archaeal sequences in both runnel ponds was identified closest to *Methanobacterium lacus*, a newly described strain that utilize H_2_/CO_2_ and methanol/H_2_ as substrates [24]. The most abundant *Methanosarcina* OTUs were 99% similar to *Methanosaeta concilii* [25], and to *Methanosarcina mazei* [26] both known to be acetoclastic methanogens. The most abundant *Methanoregula* OTU was 98% similar to *Methanoregula boonei*, a hydrogenotroph. The polygonal pond BYL1 had a high percentage of RC II and a lower percentage of *Methanosarcina* compared to the other 3 ponds.

## Discussion

The Bylot Island pro-glacial river valley's ice-wedge tundra terrain was covered by a network of ponds and was similar to the landscape of Samoylov Island, Eastern Siberia [7]. Our results clearly show that in addition to being a source of CO_2_, as opposed to a sink, runnel ponds represented a larger source of CH_4_ than polygonal ponds. Runnel ponds accounted for 44% of the open water in the valley, but contributed to 83% of the total CH_4_ emissions that included lake emissions from a 3-year diffusive rate database. Our data suggest that CH_4_ emissions from thawing permafrost could be strongly underestimated if measured only from the more frequently studied polygonal ponds [3], [5], [27], [28]. The smaller emissions from polygonal ponds may be due to more activity by the methanotroph community, and we note that stable isotopes were consistent with more CH_4_ oxidation in polygonal ponds (Fig. 3b).

Methane diffusion rates measured from runnel ponds (on average 0.76 mmol m^−2^ d^−1^) were in the same range as reported from the thermokarst lakes in Siberia [29]–[30], but relatively small compared to peatland ponds from the Hudson Bay lowland [31], which were up to 48 mmol CH_4_ m^−2^ d^−1^. However, comparing flux estimates among studies of smaller aquatic systems is difficult due to several factors that are rarely considered, such as gas collection method, gas transfer model and flux calculation, time of day, season, latitude, water body size and depth, catchment geomorphology, and finally the presence or absence of thermokarst slumping. Here, we applied a correction factor (×0.2458; see methods) based on chamber flux measurements and wind-based estimates [4] to account for the positive buoyancy flux occurring as thermal stratification evolves during the day in small-fetched and sheltered ponds. However, during the night, diffusion is likely to increase as water mixes due to heat loss, which was not included in our estimates. Moreover, estimates of CH_4_ flux with gas exchange velocity based on Fick's law, and pure diffusive gas transfer such as for CO_2_, do not take into account micro-ebullition. For these reasons, our runnel pond diffusive flux estimations may be conservative.

For the two polygonal ponds, which were measured repeatedly, the maximal ebullition rate reached 2.13 mmol m^−2^ d^−1^, decreasing by ∼1 order of magnitude over a few weeks. This was similar to the diffusive rates that were up to 0.77 mmol m^−2^ d^−1^ in the two ponds. This maximal ebullition rate was within the lowest range of values compiled by Walter et al. [30] (see their Table 1) for northern aquatic systems (their *Arctic* class), and much less than for Siberian thermokarst lakes, which reached 1563 mmol m^−2^ d^−1^. The high rates from Alaska and Siberia are from emissions categorized as point sources and hotspot ebullition, occurring in lakes with taliks and much thicker peat deposits. Taliks form under thermokarst lakes that are deep enough to have a layer of water and sediment or soil, which remains unfrozen in winter. These conditions are unlikely to occur under the shallow Bylot Island thaw ponds since they freeze to the bottom in winter, partly explaining their lower ebullition rates. Unfortunately ebullition measurements were only taken from polygonal ponds where funnels could be installed. However, considering that diffusive fluxes were on average 3.5 times higher in the runnel ponds, ebullition fluxes and overall CH_4_ production were also likely to be greater in the runnel ponds.

A larger fraction of old C would also be available for microbial degradation in the runnel ponds compared to polygonal ponds because of peat erosion down to the thickness of the ∼half meter active layer on Bylot Island. The base of the peat deposit, which is about 2 m thick, was aged at 3670±110 BP [1]. Discrete background ebullition samples collected from June to July 2011 showed little evidence of high release of this old stored C in the form of GHG from the two runnel ponds sampled. Runnel ponds however, exhibited a higher fraction of older C in CO_2_ and CH_4_ compared to polygonal ponds (Fig. 2). The utilization and release of a larger fraction of older C through point source ebullition could still occur at this site at certain times over the thaw cycle. For example, ebullition from point sources released much older C in Siberian and Alaskan thermokarst lakes, despite modern age C reported for background ebullition [15].

Permafrost peat provides substrate for aquatic microbes [32], but the preferential use of modern C recently fixed from the atmosphere could be favored because of the greater lability of this pool [17]. In the case of the cyanobacterial mat-covered polygonal ponds on Bylot Island, the negative CO_2_ flux most likely resulted from high photosynthetic rates in the mats, and the modern dates for CH_4_ suggest that abundant labile compounds coming from a modern autochthonous pool could be the main C supply for microbial activity, including methanogenesis. However, in more humic runnel ponds influenced by peat lixiviation, an older C signature in the CH_4_ than what we found was expected. The predominance of AM and the high OC content of surface sediment (1.0–25.1%) indicate that both pond types were C-rich [18]–[19]. These OC values were mostly greater than values reported in Siberian permafrost soils, for example in the Lena Delta 4–5% of OC is within the top 50 cm [33], which is similar to values from Northeast Siberia [34]. The reasons for the high OC in surface sediment of small ponds lacking taliks could be due to slow microbial degradation rates linked to seasonal re-freezing. If this were the case, then a longer melt season could result in greater CH_4_ emissions.

The sum of two methanogen genera adapted to high substrate levels was higher in runnel ponds than in polygonal ponds. These two genera have different CH_4_ production pathways, *Methanobacterium* with the HM pathway [35], and *Methanosarcina* with the AM pathway [36] (Fig. 4), suggesting community adaptability. The main methanogens in thaw ponds were *Methanosarcina*, *Methanosaeta*, *Methanobacteriaceae*, *Methanomicrobiales*, and RC II, which is similar to the community retrieved from Svalbard peatlands and wetlands [37]–[38]. Most of the descriptive studies to date on freshwater Arctic archaeal communities are from clone libraries, and at most three Orders out of the five known Orders of methanogens were found from a single site [39]–[42]. For instance, in 19 freshwater lakes, Borrel and colleagues [24] reported 468 archaeal 16S rRNA sequences from clone libraries. *Methanomicrobiales* and *Methanosarcinales* dominated these lakes, with occasional sequences belonging to the *Methanobacteriales* [43]. The higher number of methanogen Orders and presence of AM and HM pathways from Bylot Island may be a consequence of the high OC content in the ponds. Alternatively our high throughput sequencing approach with a minimum 1921 final reads per sample may have recovered the additional Orders.

Substrate availability for CH_4_ production from acetate or CO_2_ is likely to change seasonally due to the timing of ice melt and primary production, generating changes in the methanogen community structure [38]. For example, in Finnish boreal mires, there was a clear shift in the methanogen community over the arctic summer, with AM (*Methanosarcina* spp.) found only during early and mid summer [44]. On Bylot Island, both AM and HM methanogens were retrieved from the sediments. However, the isotopic signatures of CH_4_ indicated that only AM was active in July (Fig. 3) suggesting that the HM biomass had built up earlier. These results also show that AM can be a significant summer production pathway in Arctic permafrost regions, as opposed to other thermokarst systems where only HM was thought to be significant [45]. A methanogenic community composed of both AM and HM taxa will likely respond to wider temperature ranges and possible substrate changes that occur under climate stress, and both pathways should be considered in C budget estimates.

Thaw ponds contained reads with matches to methanogenic groups capable of both hydrogenotrophic and acetotrophic CH_4_ production, providing the potential for community compensation under changing ambient conditions. However, the small size and great variability in shapes, limnology and microbial ecology of the ponds represent a challenge for scaling up their importance for global C cycling, especially since these ponds are primarily found in remote regions where logistic constraints are great. But considering that they have the potential to develop in permafrost and glaciated-influenced landscapes covering 9.6 millions of km^2^ in circumpolar regions [23], these small systems certainly deserve more attention. As the Arctic warms and permafrost recedes, the abundance of tundra ponds, especially runnel ponds generated by thaw slumping, is likely to increase. The higher CH_4_ emissions measured from runnel ponds, and their potential to contain organic carbon deposited thousands of years ago qualify them as a positive feedback system contributing to climate dynamics.

## Methods

### Study Site

Samples were collected at Sirmilik National Park, Bylot Island, Nunavut (73°09′N, 79°58′W; Fig. 1) in the continuous permafrost region of Canadian Arctic, with an active layer depth between 40 and 60 cm (D. Fortier, pers. comm.). Required permits to carry out sampling were approved by the Parks of Canada Agency for Sirmilik National Park (Research and Collection Permit) and the Nunavut Research Institute (Nunavut Science License). The Bylot SILA station recorded a mean annual air temperature of −14.5°C, with summer temperatures from June to August averaging 4.5°C and winter temperatures from December to January averaging −32.8°C. Precipitation between June and August (1994–2007) was about 94 mm. Thaw ponds and lakes covered 4.2% of the ∼65 km^2^ pro-glacial river valley in 2010, as obtained from a high resolution image from WorldView-1, with runnel ponds contributing approximately 44% of the surface water compared to polygonal ponds contributing 27%. Polygonal ponds form on top of low centered peat polygons, and are generally 0.5 to 1.5 m deep with an area <500 m^2^. Runnel ponds form over melting ice wedges, and are often shallower than 0.5 m but sometimes form long networks (Fig. 1). Both pond types freeze to bottom in winter, and are unfrozen for approximately 110 days per year. The sum of all daily temperatures above freezing averaged 447 thawing-degree days (http://www.cen.ulaval.ca/bylot/climate-description-bylotisland.htm).

### Sampling

In July 2009, 34 ponds were sampled for dissolved GHG concentrations and flux estimation, with 19 sampled for carbon and hydrogen stable isotope ratios (Table 2), and 4 ponds (2 polygonal and 2 runnel ponds) for archaeal diversity assessment via pyrosequencing (BYL1, 22, 24, 27; Table 1). These 4 ponds were selected to represent a range of physiochemical properties, which were measured in previous years. In July 2010, 14 ponds from the 2009 series were re-sampled for dissolved GHG concentrations and flux estimations. In 2011, from mid June to mid July, dissolved GHG concentrations and flux estimates were obtained from a total of 43 ponds, including 15 from the 2009 series. In addition, ebullition samples were taken from 4 ponds (2 polygonal ponds; BYL1 and 80 and 2 runnel ponds; BYL27 and 38) for stable isotopes and Δ^14^C analysis, and ebullition rates were measured from the two polygonal ponds, which were deep enough to install the funnels needed for rate measurements. Ebullition flux was not measured from runnel ponds because the funnels were too wide to be correctly installed in the shallow and narrow ponds, exemplifying the difficulties in Arctic sampling. Also in 2011, GHG dissolved flux was measured approximately every 2 h over 26 h on 2 polygonal ponds (BYL1 and BYL80) to examine the daily variations in GHG dissolved concentrations.

**Table 2:** Compilation of thaw ponds samples collected each year.

		Year	Number of ponds	Methods
**Dissolved GHG**	GHG flux	2009–2011	33P, 58R	Dissolved concentrations
	Production pathway	2009	9P, 10R	Stable isotopes
	Diurnal variations	2011	2P	Hourly flux measures
**Ebullition**	Ebullition flux	2011	2P	Funnel traps
	Production pathway	2011	2P, 2R	Stable isotopes
	C-source (age)	2011	2P, 2R	^14^C dating
**DNA**	Methanogens	2009	2P, 2R	Pyrosequencing
**Environment**	Limnology	2009	2P, 2R	Nutrients, ions, pH, temp, O_2_, DOC
	C-source (amount)	2011	9P, 8R	Sediment OC

Polygonal ponds (P); runnel ponds (R); dissolved organic carbon (DOC); organic carbon (OC); Greenhouse gases (GHG, including CO_2_ and CH_4_); ebullition is GHG released as bubbles; production pathway indicates CH_4_ produced by acetoclastic methanogenesis (AM) or hydrogenotrophic methanogenesis (HM); temperature (temp). Note that most samples were collected in 2009, with diurnal, ebullition and sediment OC collected in 2011, which was the only occasion when appropriate sampling gear was available.

### Limnological characteristics

Surface water pH was measured with a 600R multi-parametric probe (Yellow Spring Instrument). The surface temperatures of one polygonal (BYL1) and one runnel pond (BYL24) were continuously recorded from July 2008 to July 2009 (thermistors, HOBOware™ U12, Onset). Pond water filtered through 0.2 µm pre-rinsed cellulose acetate filters (Advantec) was used for dissolved organic carbon (DOC) concentrations (Shimadzu TOC-5000A carbon analyzer calibrated with potassium biphthalate). Soluble reactive phosphorus (SRP) and major ions were measured on filtered samples [4]. Unfiltered water samples were fixed with H_2_SO_4_ (0.15% final concentration) for total phosphorus (TP) and total nitrogen (TN) quantification as in [46]. In 2011, 5 mL of surface sediment were collected for total organic carbon content (TOC) and processed with 0.1 mol L^−1^ of sulfuric acid on an elemental analyzer (CHNS-932, LECO Instruments) [47].

### Diffusive flux

Dissolved CO_2_ and CH_4_ concentrations in surface waters were obtained by equilibrating 2 L of water with 20 mL of ambient air for 3 minutes. Most sampling occurred between 9 am and 4 pm. The resulting headspace was injected into glass vials (BD 3 mL Vacutainers, or Labco 5.9 mL Exetainers), helium flushed and vacuumed [48]. Samples were analyzed by gas chromatography (Varian 3800 with COMBI PAL head space injection, CP-Poraplot Q 25 m 3 0.53 mm column, flame ionization detection), and dissolved gas concentration calculated using Henry's Law:
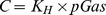
where K_H_ is Henry's constant adjusted according to ambient water temperature, and pGas is the partial pressure of CO_2_ or CH_4_ in the headspace. Dissolved GHG flux (F_d_) was calculated as:
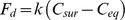
where C_sur_ is the gas concentration in surface water, C_eq_ is the gas concentration when in equilibrium with the atmosphere at ambient temperature (global atmospheric concentrations were used), and k is the gas exchange velocity calculated as:
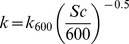
where Sc is the Schmidt number calculated from empirical third-order polynomial fit to water temperature and corrected at 20°C. The gas exchange coefficient k_600_ of Cole and Caraco [49] was used as a first approximation:
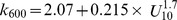
where U_10_ is the wind speed at 10 m above ground. However, this gas transfer model is not adequate for small aquatic systems (small fetched) where turbulence is controlled by heat exchange rather than wind [50]. Therefore, a correction factor (×0.2458) was applied, obtained from a series of simultaneous CO_2_ flux measurements from a floating chamber connected to an EGM-4 (PP-Systems) performed at the same time as surface gas concentrations were collected [4] (data from 2007 to 2010, n = 57, r^2^ = 0.689, p<0.001; unpubl. data).

### Ebullition

Ebullition flux and bubble characterization (composition, δ^13^C and δD, and ^14^C dating; see below) were obtained from submerged funnels. Bubble samples were collected in pre-combusted (500°C for 2 h), miliQ-rinsed, 125 mL glass bottles, helium flushed and vacuumed, with butyl rubber caps. Funnels were installed in polygonal ponds BYL1 and 80 from 18 June to 13 July 2011. Ice was present at the bottom of BYL80 from 18 to 22 June, while no ice was present in BYL1. Ebullition flux (F_e_) was obtained from passive accumulation of gas in funnels, and calculated as:
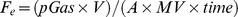
where V is the gas volume collected, A is the funnel area (0.3526 m^2^), and MV the gas molar volume at ambient air temperature. In addition, gas was collected from 22 to 26 June from stirred sediments for stable isotopes and ^14^C dating in ponds BYL27 and 38, since ebullition rate did not provide sufficient gas and funnels were too large for proper installation in shallow and narrow runnel ponds.

### Stable isotopes

Stable isotopes of C and H in CO_2_ and CH_4_ (δ^13^CO_2_, δ^13^CH_4_, and δD_CH4_) were analyzed at the Biochemistry Laboratory of the School of Earth and Ocean Sciences (University of Victoria, Canada). Gas samples in Wheaton bottles were analyzed for δ^13^CH_4_ by introducing the gas onto a GSQ PLOT column (0.32 mm ID, 30 m) using a Valco 6-port valve and sample loop. After chromatographic separation, the CH_4_ passes through an oxidation oven (1030°C), a Nafion water trap, and open-split interface to a Continuous Flow-Isotope Ratio Mass Spectrometer (CF-IRMS). The δ^13^CO_2_ was measured similarly, but without the combustion oven. Precision for the δ^13^CH_4_ and δ^13^CO_2_ analyses was ±0.2‰. Hydrogen isotope ratios of CH_4_ (δD_CH4_) were measured by a TC/EA pyrolysis unit (1450°C) interfaced to a CF-IRMS. Precision for the δD_CH4_ analyses was ±3‰, relative to VSMOW.

### Δ^14^C analysis

Methane and CO_2_ were separated by a continuous flow line consisting of purification and combustion traps [51] as follows: first, CO_2_ was frozen in liquid nitrogen (LN_2_), second, carbon monoxide (CO) was oxidized to CO_2_ in a 300°C CuO furnace and frozen in a second LN_2_ trap, finally, non-condensable CH_4_ was oxidized to CO_2_ in a CuO furnace at 975°C (Lindberg/Blue M Tube Furnace, Thermo Scientific). The resulting CO_2_ and H_2_O from CH_4_ combustion were further separated cryogenically on the vacuum line. Purified CO_2_ was graphitized using the sealed tube zinc reduction method [52]. The ^14^C analysis was conducted at the Keck Carbon Cycle AMS (KCCAMS) facility at the University of California, Irvine (UCI), on a compact accelerator mass spectrometer (AMS) system from National Electrostatics Corporation (NEC 0.5MV 1.5SDH-2 AMS), with a modified NEC MC-SNIC ion-source [53]–[54]. The in-situ simultaneous AMS δ^13^C measurement allowed for fractionation corrections occurring inside the AMS system and during graphitization, significantly improving the precision and accuracy, with a day-to-day analysis relative error of 2.5 to 3.1‰ based on secondary standards, and including extraction, graphitization and AMS measurement.

### Archaeal diversity

Surface water was filtered sequentially through a 3 µm pore size polycarbonate filter and a 0.2 µm Sterivex unit (Millipore). Filters were immersed in buffer (40 mM EDTA; 50 mM Tris at pH 8.3; 0.75 M sucrose), stored in liquid nitrogen in the field (≤2 weeks), and then stored at −80°C until extraction. Cellular DNA was extracted from both filters, with a phenol∶chloroform∶Indole-3-Acetic Acid (25∶24∶1) and chloroform∶Indole-3-Acetic Acid (24∶1) separation and DNA quantified by spectrophotometry (Nanodrop ND-1000). Surface sediment samples were collected using a cut 60 mL sterile plastic syringe to depth of around 6.5 cm, placed in sterile plastic bags and homogenized. A sub-sample of 3 mL was squeezed from the bag into 5 mL cryotubes with buffer and stored as above. DNA was extracted using the MO BIO Kit (RNA Powersoil total RNA isolation kit #12866-25 and DNA elution accessory kit #12867-25) allowing both RNA and DNA to be extracted at the same time, but only DNA was sequenced for this study. Once extracted the DNA was quantified as above.

A PCR reaction mixture of 1× HF buffer (NEB), 200 µM dNTP (Feldan Bio), 0.4 mg mL^−1^ BSA (Fermentas), 0.2 µM of each 454 primer (969F: ACGCGHNRAACCTTACC and 1401R: CRGTGWGTRCAAGGRGCA) [55], 1 U of Phusion High-Fidelity DNA polymerase (NEB), and 0.1–1 µL of template DNA for sediment samples, or 2 µL for water samples. Three separate DNA concentrations were used for each sample, from 1× to 2.22×, to reduce PCR bias. Amplification cycles included denaturing at 98°C for 30 s, 30 cycles of denaturing at 98°C for 10 s, annealing at 55°C for 30 s, extension at 72°C for 30 s, and a final extension at 72°C for 5 min. For each sample, the triplicate reactions were pooled together for purification (QIAquick PCR purification kit; QIAGEN) and quantification (Nanodrop ND-1000). The resulting sample coded amplicons were mixed in equal proportions and sequenced on a Roche 454 GS-FLX Titanium platform at Université Laval Plateforme d'analyses Génomiques. Raw reads were submitted to NCBI Sequence Read Archive (SRA) under the accession number SRA039814, with a Sequence Read Experiment (SRX) number SRX319084. Resulting reads were subjected to pyrotag pre-processing, quality control, and taxonomic analyses [55]. Low-quality reads were identified and removed if they contained any non assigned nucleotides (N's), were <150 bp not including the adaptor and sample tag-code, if they exceed the expected amplicon size, and if the Forward primer sequence was incorrect. The remaining reads were then trimmed if there were nucleotide bases after the reverse primer. Next, reads were aligned using mother [56]–[57] against SILVA reference alignments, and then manually checked to remove misaligned reads. The number of reads after processing ranged from 1921 to 2105, and for downstream analysis was randomly resampled to 1921 reads. The SILVA database (version 108) was used for archaeal identifications, including additional previously generated clone library sequences [41], [58]–[59] from the C. Lovejoy laboratory.

## Supporting Information

File S1 **Figure S1, Seasonal melting and freezing.** Surface water temperature for one polygonal pond (BYL1) and one runnel pond (BYL24), from July 2008 to July 2009. **Figure S2, Diffusive greenhouse gas flux from polygonal and runnel ponds.** Data collected from summer 2009, 2010 and 2011, including 33 measurements from polygonal ponds and 58 from runnel ponds. The diffusive flux was calculated using the wind-based model of Cole and Caraco [41], but estimations were corrected with a regression equation comparing floating chamber CO_2_ flux to wind-based flux (see Methods). **Table S1, Methane emission ranges (median value in parenthesis) through diffusion (N = 4) and ebullition (N = 8) from two polygonal ponds (BYL1 and BYL80), and diffusive flux from 12 other polygonal ponds and 14 other runnel ponds located on the same site measured from 18 June to 16 July 2011.** **Table S2, Range (median) of δ^13^CO_2_, δ^13^CH_4_, and δD_CH4_ values for diffusion and ebullition gas samples, also given separately for polygonal and runnel thaw ponds.**
(PDF)
