# Supplementary material for: Small Thaw Ponds: An Unaccounted Source of Methane in the Canadian High Arctic
Source: PLoS One. 2013 Nov 13;8(11):e78204. doi: 10.1371/journal.pone.0078204 (PMC3827239; doi:10.1371/journal.pone.0078204)

Supplementary, Table S1. Methane emission ranges (median value in parenthesis) through diffusion (N=4) and ebullition (N=8) from two polygonal ponds (BYL1 and BYL80), and diffusive flux from 12 other polygonal ponds and 14 other runnel ponds located on the same site measured from 18 June to 16 July 2011.

	**Diffusive flux**[1]	**Ebullition flux**[2]
	**mmol m^{-2} d^{-1}**	
**BYL1**	0.04 – 0.37 (0.10)	0.01 – 0.07 (0.04)
**BYL80**	0.05 – 0.77 (0.08)	0.18 – 2.13 (0.91)
**POLYGONAL PONDS**	0.02 – 0.25 (0.06)	–
**RUNNEL PONDS**	0.02 – 0.98 (0.30)	

[1]Diffusive flux was calculated using the wind-based model of Cole and Caraco[41], but estimations were corrected with a regression equation comparing floating chamber CO_2 flux to wind-based flux (see Methods).

[2]Ebullition flux was calculated from the gas collected with a submerged funnel over periods of 21 to 121 h.

Supplementary, Table S2. Range (median) of $\delta^{13}CO_2$, $\delta^{13}CH_4$, and δD_{CH4} values for diffusion and ebullition gas samples, also given separately for polygonal and runnel thaw ponds.

	**$^{13}CO_2$ (‰ vs. VPDB)**	**$^{13}CH_4$ (‰ vs. VPDB)**	**D_{CH4} (‰ vs. VSMOW)**
**Diffusion (n=17)**	-36.6 to -12.7<br>(-17.9)	-59.8 to -39.9<br>(-48.6)	-368.5 to -304.14<br>(-342.30)
**Polygonal (n=7)**	-21.4 to -15.4<br>(-18.3)	-53.8 to -41.5<br>(-47.2)	-347.75 to -304.17<br>(-330.94)
**Runnel (n=10)**	-36.6 to -12.7<br>(-17.6)	-59.8 to -39.9<br>(-50.0)	-368.50 to -319.53<br>(-350.24)
**Ebullition (n=10)**	-16.1 to -0.1<br>(-7.2)	-61.7 to -54.8<br>(-58.8)	-408.8 to -338.1<br>(-372.9)
**Polygonal (n=7)**	-8.8 to -0.1<br>(-4.8)	-61.7 to -57.4<br>(-60.0)	-381.7 to -338.1<br>(-363.1)
**Runnel (n=3)**	-16.1 to -10.0<br>(-13.0)	-57.3 to -54.8<br>(-55.9)	-408.8 to -388.4<br>(-396.0)

Figure S1. Seasonal melting and freezing: Surface water temperature for one polygonal pond (BYL1) and one runnel pond (BYL24), from July 2008 to July 2009.

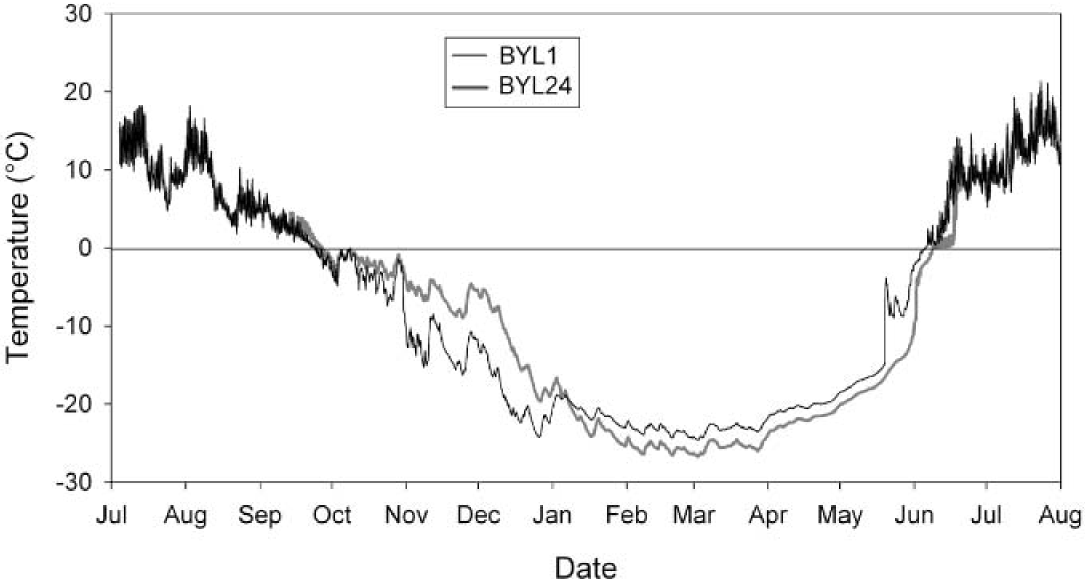

Figure S2. **Diffusive greenhouse gas flux from polygonal and runnel ponds**: Data collected from summer 2009, 2010 and 2011, including 33 measurements from polygonal ponds and 58 from runnel ponds. The diffusive flux was calculated using the wind-based model of Cole and Caraco [41], but estimations were corrected with a regression equation comparing floating chamber CO_2 flux to wind-based flux (see Methods).

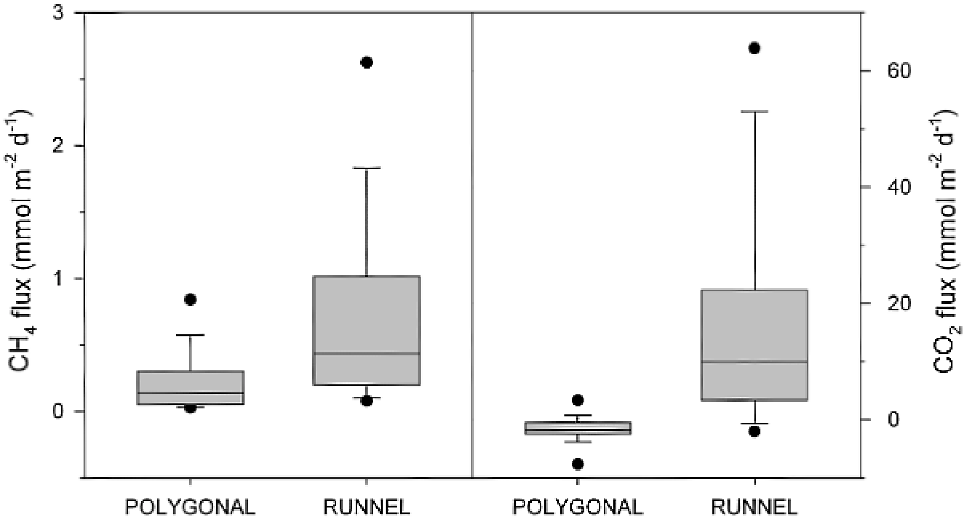

Supplement: File S1 — Figure S1, Seasonal melting and freezing. Surface water temperature for one polygonal pond (BYL1) and one runnel pond (BYL24), from July 2008 to July 2009. Figure S2, Diffusive greenhouse gas flux from polygonal and runnel ponds. Data collected from summer 2009, 2010 and 2011, including 33 measurements from polygonal ponds and 58 from runnel ponds. The diffusive flux was calculated using the wind-based model of Cole and Caraco [41], but estimations were corrected with a regression equation comparing floating chamber CO2 flux to wind-based flux (see Methods). Table S1, Methane emission ranges (median value in parenthesis) through diffusion (N = 4) and ebullition (N = 8) from two polygonal ponds (BYL1 and BYL80), and diffusive flux from 12 other polygonal ponds and 14 other runnel ponds located on the same site measured from 18 June to 16 July 2011. Table S2, Range (median) of δ13CO2, δ13CH4, and δDCH4 values for diffusion and ebullition gas samples, also given separately for polygonal and runnel thaw ponds. (PDF) [file pone.0078204.s001.pdf]
